# Optimizing Doses of Ceftolozane/Tazobactam as Monotherapy or in Combination with Amikacin to Treat Carbapenem-Resistant *Pseudomonas aeruginosa*

**DOI:** 10.3390/antibiotics11040517

**Published:** 2022-04-13

**Authors:** Worapong Nasomsong, Parnrada Nulsopapon, Dhitiwat Changpradub, Supanun Pungcharoenkijkul, Patomroek Hanyanunt, Tassanawan Chatreewattanakul, Wichai Santimaleeworagun

**Affiliations:** 1 Division of Infectious Diseases, Department of Internal Medicine, Phramongkutklao Hospital and College of Medicine, Bangkok 10400, Thailand; nasomsong.w@gmail.com (W.N.); dhitiwat@yahoo.com (D.C.); 2 Department of Pharmacy, Faculty of Pharmacy, Silpakorn University, Nakhon Pathom 73000, Thailand; aom.olivegreen1812@gmail.com; 3 Pharmaceutical Initiative for Resistant Bacteria and Infectious Diseases Working Group [PIRBIG], Faculty of Pharmacy, Silpakorn University, Nakhon Pathom 73000, Thailand; 4 Pharmacy Unit, Nopparat Rajathanee Hospital, Bangkok 10230, Thailand; supanun.pung@gmail.com; 5 Division of Microbiology, Department of Clinical Pathology, Phramongkutklao Hospital, Bangkok 10400, Thailand; patomroek.han@pcm.ac.th (P.H.); joymicro14@gmail.com (T.C.)

**Keywords:** antibiotic combination, minimum inhibitory concentration, Monte Carlo, synergistic effect

## Abstract

Carbapenem-resistant *Pseudomonas aeruginosa* (CRPA) is a hospital-acquired pathogen with a high mortality rate and limited treatment options. We investigated the activity of ceftolozane/tazobactam (C/T) and its synergistic effects with amikacin to extend the range of optimal therapeutic choices with appropriate doses. The E-test method is used to determine in vitro activity. The optimal dosing regimens to achieve a probability of target attainment (PTA) and a cumulative fraction of response (CFR) of ≥90% were simulated using the Monte Carlo method. Of the 66 CRPA isolates, the rate of susceptibility to C/T was 86.36%, with an MIC_50_ and an MIC_90_ of 0.75 and 24 µg/mL, respectively. Synergistic and additive effects between C/T and amikacin were observed in 24 (40%) and 18 (30%) of 60 CRPA isolates, respectively. The extended infusion of C/T regimens achieved a ≥90% PTA of 75% and a 100% *f*T > MIC at C/T MICs of 4 and 2 µg/mL, respectively. Only the combination of either a short or prolonged C/T infusion with a loading dose of amikacin of 20–25 mg/kg, followed by 15–20 mg/kg/day amikacin dosage, achieved ≥90% CFR. The C/T infusion, combined with currently recommended amikacin dose regimens, should be considered to manage CRPA infections.

## 1. Introduction

*Pseudomonas aeruginosa* is a significant cause of hospital-acquired infections and is frequently multidrug-resistant (MDR) [1]. MDR *P*. *aeruginosa* was reported in 14.7% and 22.0% of cases of bloodstream infections and pneumonia, respectively. The high mortality rate of infections with MDR *P.* *aeruginosa* makes treating serious infections more challenging. Furthermore, MDR *P. aeruginosa* is frequently resistant to carbapenems and other β-lactams. β-lactam resistance is mediated via multiple mechanisms, including the acquisition of metallo-β-lactamases, the increased production of chromosomal AmpC, increased efflux, and changes in membrane permeability [2,3]. The most consistently active drugs against MDR *P.* *aeruginosa* are aminoglycosides and polymyxins, but pharmacokinetic limitations and their association with worse outcomes when given as monotherapy make their use suboptimal [4,5].

Ceftolozane is a novel cephalosporin with enhanced activity against *P*. *aeruginosa*. When combined with the well-described β-lactamase inhibitor tazobactam, ceftolozane/tazobactam (C/T) is a safe and effective treatment for complicated urinary tract infections and complicated intraabdominal infections (in combination with metronidazole) caused by Gram-negative organisms, including *P*. *aeruginosa*. C/T also has good in vitro activity against MDR *P*. *aeruginosa* [6]. Relative to most β-lactams, ceftolozane has improved activity against *P. aeruginosa* because it is stable against AmpC enzymes produced by *P. aeruginosa*, is unaffected by active efflux, and is not appreciably affected by porin channel changes. Tazobactam protects ceftolozane from destruction by most extended-spectrum β-lactamases, but does not add to its activity against *P. aeruginosa* [7,8].

Shortridge et al. described the excellent activity of C/T against 3851 isolates of *P*. *aeruginosa*, including MDR or extensively drug-resistant (XDR) isolates. As the most active β-lactam agent tested against *P. aeruginosa*, C/T may be an important agent to treat severe bacterial infections [9]. Additionally, antipseudomonal antibiotics, such as aztreonam, amikacin, and meropenem, produce a synergistic effect with C/T [10,11,12]. However, among the few reports of the synergistic effect of C/T with other antibiotics, studies involving isolates of *P*. *aeruginosa* are limited.

Treatment failure may occur in critically ill patients infected with MDR or XDR *P*. *aeruginosa*. A minimum inhibitory concentration (MIC) of >2 µg/mL for C/T was recently reported as being associated with 30-day mortality in patients infected with MDR *P*. *aeruginosa* treated with the approved recommended dosage regimens [13]. Furthermore, the pharmacokinetic/pharmacodynamic (PK/PD) properties of antibiotics become altered due to pathophysiological changes in critically ill patients. Thus, PK/PD analysis using Monte Carlo simulation is used to design optimal antibiotic dosing regimens, thereby maximizing antibiotic activity, increasing the probability of clinical success, and reducing the rate of antibiotic resistance [14,15].

As described above, C/T seems an attractive agent to treat infections with carbapenem-resistant *P. aeruginosa* (CRPA). However, in vitro susceptibility data of C/T against CRPA in Thailand has never been reported. Thus, this study investigated the in vitro activity of C/T and the synergistic effect of C/T and amikacin against CRPA isolates from clinical specimens of patients at Phramongkutklao Hospital, Thailand, to assess the optimal dosing regimens of C/T as a monotherapy or in combination with amikacin in critically ill patients.

## 2. Results

### 2.1. In Vitro Susceptibility of C/T and Comparator Agents

Of the 66 CRPA strains isolated from unique patients, 55 (83.33%) were MDR, 9 (13.64%) were XDR, and 2 (3.03%) were PDR. The susceptibility test results of C/T and other comparator agents are presented in Table 1 and Table 2.

According to broth microdilution (BMD), the CRPA strains were most susceptible to colistin (96.97%), amikacin (89.39%), and gentamicin (83.33%), and only 4.55% and 13.64% were susceptible to imipenem and meropenem, respectively. Regarding other antipseudomonal β-lactams, the susceptibility rates of CRPA were 63.64%, 63.64%, and 45.45% to ceftazidime, cefepime, and piperacillin/tazobactam, respectively. Among non-β-lactams, amikacin and gentamicin showed good activity against CRPA.

C/T was the most active β-lactam agent against the 66 strains of CRPA from the E-test method, of which 86.36% showed susceptibility at an MIC_50_ of 0.75 µg/mL and an MIC_90_ of 24 µg/mL.

### 2.2. Synergistic Activities

Synergistic, additive, and indifference effects of C/T and amikacin were observed in 24 of 60 (40%), 18 of 60 (30%), and 18 of 60 (30%) CRPA isolates (Table 2 and Table 3). No antagonistic activity was observed between the combination of C/T and amikacin.

### 2.3. PTA and CFR

The PTA, including 75% and 100% *f*T > MIC for ceftolozane and 20% *f*T ≥ 1 µg/mL for tazobactam, for each dosing regimen at specific MICs is shown in Table 4 and Table 5. All antibiotic dosing regimens (detailed in Section 4.4) met the criteria of ≥90% PTA of 20% *f*T ≥ 1 µg/mL for tazobactam. The PTA of achieving 75% *f*T > MIC following ceftolozane administration by a 0.5-h infusion, a prolonged 4-h infusion, and a loading dose followed by continuous infusion was 93.92%, 93.61%, and 93.61% at MICs of 2, 4, and 8 µg/mL, respectively. Furthermore, the PTA of achieving 100% *f*T > MIC following ceftolozane administration by a 0.5-h infusion, a prolonged 4-h infusion, and a loading dose followed by continuous infusion was 95.50%, 94.75%, and 93.60% at MICs of 1, 2, and 8 µg/mL, respectively. None of the C/T dosing regimens achieved the PTA target when the MIC was ≥16 µg/mL.

For C/T, only combination regimens achieved the target of ≥90% CFR at 75% and 100% *f*T > MIC. None of the amikacin dosing regimens achieved the CFR target when administered as monotherapy. Fortunately, when amikacin was combined with C/T, the amikacin dosage, a loading dose of 20–25 mg/kg, followed by 15–20 mg/kg every 24 h, met the CFR target. The CFR for each dosing regimen of C/T, amikacin, and its combination are presented in Table 6 and Table 7.

## 3. Discussion

There are limited treatment options for CRPA infections. The agents to which CRPA is susceptible include colistin or polymyxin B, which exhibit a suboptimal treatment outcome and a high rate of adverse reactions, especially nephrotoxicity. Our study in a Thai university hospital demonstrated the attractive in vitro activity of C/T against CRPA, with a susceptibility rate of 86.36%, which correlated with other studies, where the susceptibility rate of MDR *P*. *aeruginosa* or CRPA ranged from 67.2–85.9% [9,16]. Although C/T is considered the most active β-lactam against both susceptible and resistant strains of *P*. *aeruginosa*, a study from Singapore revealed a susceptibility rate of 37.9% for CRPA to C/T. This discrepancy was mainly attributed to the presence of carbapenemase-producing isolates, particularly metallo-β-lactamases [17].

Due to the high rate of susceptibility of CRPA to C/T, C/T may be useful as an empirical or definite antibiotic therapy for the treatment of infections suspected or known to be caused by CRPA. A clinical study showed that C/T was successful in treating 71% of patients with MDR *P*. *aeruginosa* infections [18]. Therefore, a polymyxin-sparing strategy may reduce antibiotic-related adverse events, as well as minimize the over-prescription of polymyxin.

Among the few reports of the synergistic effect of C/T with other antibiotics, studies involving isolates of *P*. *aeruginosa* are limited. Antipseudomonal antibiotics that produce a synergistic effect with C/T include aztreonam, amikacin, and meropenem [10,11,12]. In our study, we observed 40% synergism between C/T and amikacin against CRPA isolates using E-test methods. A recent study from Greece performed synergistic testing between C/T and amikacin against MDR *P. aeruginosa* using a time-kill assay, and a synergistic effect was observed in 85% [19]. These differences and discordant results may be due to several factors. First, the agreement between the time-kill assay and E-test crossing method ranged from 3–71% in MDR Gram-negative bacilli, including *P. aeruginosa* [20]. Second, differences in the genotypic resistance of bacterial strains may affect the synergistic result [19]. The good bactericidal and synergistic activity observed with the combination of C/T and amikacin may be attributed to β-lactam-mediated membrane damage, leading to increased aminoglycoside uptake [21]. Clinical data regarding the treatment outcome of C/T combination therapy against MDR Gram-negative bacilli, mostly MDR *P. aeruginosa*, revealed a significant decrease in mortality. A systematic review and meta-analysis included 8 non-randomized studies of C/T for treatment as monotherapy and combination therapy. The results showed that C/T in combination was associated with clinical improvement (OR, 0.97; 95% CI, 0.54 to 1.74; *p* = 0.954) and statistically lower mortality at 30 days (OR, 0.31; 95% CI, 0.10 to 0.97; *p* = 0.045) than the patient receiving C/T monotherapy [22]. Furthermore, a successful treatment outcome and rapid microbiological clearance using combination therapy of C/T with tobramycin against C/T-resistant *P. aeruginosa* in a severely neutropenic patient were also reported [23]. Thus, C/T combination therapy is potentially beneficial when combating refractory infections of MDR *P. aeruginosa*. Aminoglycosides, particularly amikacin, should be considered as the combination agent with C/T to achieve a good synergistic or additive effect.

The magnitude of the PK/PD target for cephalosporins that provided a maximal bactericidal effect was reported to range from 60–70% *f*T > MIC in preclinical studies, whereas the magnitude of the cephalosporin PK/PD target to achieve a clinical cure and a microbiological cure was reported as 100% and 60–100% *f*T > MIC, respectively, in clinical studies [24]. When using ceftolozane against *P. aeruginosa*, the % *f*T > MIC to achieve a 1- or 2-log reduction ranged from 30–40% [25,26]. Therefore, using 75% and 100% *f*T > MIC as the PK/PD targets of ceftolozane in the simulated regimens may be adequate to predict the maximum bactericidal effect and microbiological cure.

The simulation studies revealed that antibiotic dosing regimens by prolonged infusion or continuous infusion had greater potential than intermittent infusion. Prolonged infusion or continuous infusion regimens achieved a target of ≥90% PTA of 75% and 100% *f*T > MIC with C/T MICs ≤ 4 µg/mL (CLSI susceptible breakpoint) and ≤2 µg/mL, respectively, whereas a 1.5 g intermittent infusion every 8 h (an approved C/T dosing regimen for complicated urinary tract and intraabdominal infections) achieved ≥90% PTA at C/T MICs of ≤2 and ≤1 µg/mL, respectively, which agreed with previous studies [27,28]. There are concerns about the stability of C/T when using prolonged infusion or continuous infusion regimens. However, it was recently reported that C/T is stable for at least 24 h in 0.9% normal saline and 5% glucose solution in real-world conditions when stored in polypropylene tubes at room temperature (22 °C) without light exposure [29]. Therefore, the extended infusion of C/T is feasible, and because it increases the probability of treatment success, it may be a recommended regimen for the treatment of infections.

The synergistic effects of C/T plus amikacin contributed to achieving a target of ≥90% CFR. None of the prolonged or continuous infusions of C/T monotherapy achieved the target CFR. A previous study showed that only a continuous infusion of C/T monotherapy met the target of ≥85% CFR at 40%, 60%, and 100% *f*T > MIC [28]. However, when C/T combined with amikacin, all C/T dosing regimens except intermittent regimens and all amikacin dosing regimens (loading dose 20–25 mg/kg, followed by 15–20 mg/kg/day) reached the CFR target of ≥90%. In 2017, Kato et al. recommended that the amikacin initial dose required to achieve C_max_/MIC ≥ 8 was 15 mg/kg/day, and the amikacin maintenance dosage was 15 mg/kg/day at amikacin MICs ≤ 4 µg/mL [30]. Fortunately, all clinical CRPA isolates except no.47 had amikacin MICs ≤ 4 µg/mL when combined with C/T. If synergism occurs, the MIC values of each antibiotic were reduced by at least 1-fold dilution. The decrease in MIC affects an increase *f*T > MIC for C/T and C_max_/MIC for amikacin, respectively, resulting in a greater achievement of the probability of CFR target in each antibiotic. Thus, it may be advantageous to consider C/T plus amikacin as an empirical therapy.

To our knowledge, this is the first study to determine the in vitro susceptibility and synergistic effect of C/T against CRPA isolates in Thailand. However, several limitations were encountered. First, the BMD method, which is the gold standard of antimicrobial susceptibility testing, was not performed for C/T. However, the E-test method for C/T is a simple and acceptable method for susceptibility testing [31]. Second, the E-test crossing method was selected as the synergistic testing method in this study. It is widely used in clinical practice because it is easy to perform. However, the other methods for synergistic study, especially time-kill assay as the gold standard, can be evaluated. Third, the genotypic resistance characteristics of the CRPA isolates were not investigated. Thus, the molecular basis of the characteristics of these CRPA strains, which might contribute to a better understanding the results, were not explored. Fourth, this was a limited single- center study, which may affect the generalizability of the CFR results. Thus, our findings should be appraised and compared with other cohorts. Furthermore, we recommend that a nationwide multicenter study using standardized antimicrobial susceptibility testing methods based on various types of CRPA should be undertaken. Fifth, the antibiotic dosing regimens were simulated using typical pharmacokinetic parameters [28]. Thus, antibiotic dosing regimens for C/T and for amikacin based on creatinine clearance should be determined and monitored by the therapeutic drug monitoring (TDM) for amikacin in order to be more effective and less nephrotoxic. Finally, our clinical CRPA isolates were mostly susceptible to amikacin; thus, the optimal amikacin dosing recommendation in the combination therapy should be used if any CRPA isolates are susceptible to amikacin. Despite these limitations, this study provides essential information for treating *P. aeruginosa* in clinical practice, particularly CRPA, where treatment options are extremely limited. In summary, C/T had a fair synergistic effect with amikacin and may be considered as a combination therapy in CRPA infection.

## 4. Materials and Methods

### 4.1. Bacterial Identification and Antimicrobial Susceptibility Test

From January–December 2020, CRPA isolates were collected from patients by the microbiology laboratory at Phramongkutklao Hospital, a 1200-bed teaching hospital of the Phramongkutklao College of Medicine, Royal Thai Army, Bangkok, Thailand. All studied CRPA isolates were identified as *P. aeruginosa* using matrix-assisted laser desorption ionization time-of-flight mass spectrometry.

Antimicrobial susceptibility tests were determined by the standard BMD method using frozen 96-well plates (THAN1F, Sensititre^TM^, Thermo Fisher Scientific, Waltham, MA, USA). The colonies were picked up, suspended in distilled water, and adjusted the turbidity to 0.5 McFarland. Next, 10 µL of the prepared bacterial suspension (~10^8^ cfu/mL) was diluted in cation-adjusted Mueller Hinton Broth (Sensititre^TM^ cation-adjusted Mueller Hinton Broth with TES; TREK Diagnostic Systems Ltd., East Grinstead, West Sussex, UK) at ~1:1000 dilution. The final bacterial suspension for testing contained an inoculum density of ~10^5^ cfu/mL. The antimicrobial concentrations tested were as follows: amikacin, 8–32 μg/mL; cefepime, 1–32 μg/mL; ceftazidime, 1–32 μg/mL; ciprofloxacin, 0.06–2 μg/mL; colistin, 1–4 μg/mL; gentamicin, 2–8 μg/mL; imipenem, 0.5–8 μg/mL; meropenem, 0.5–8 μg/mL; piperacillin/tazobactam, 8/4–64/4 μg/mL; levofloxacin 0.06–8 μg/mL. The clinical isolates of *P*. *aeruginosa* were considered carbapenem-non-susceptible if their MIC values for meropenem or imipenem were ≥4 μg/mL [32].

The clinical isolates of CRPA were selected and underwent antimicrobial susceptibility testing against C/T and amikacin. The MIC values of each studied antibiotic were determined using E-test strips (Liofilchem, Inc., Waltham, MA, USA). A purified colony of isolated strains was picked up and suspended to 0.9% in normal saline (Univar^®^, Ajax Finechem Pty Ltd., Taren Point, Australia) as 0.5 McFarland standard. The susceptibility data of C/T and amikacin against the CRPA isolates were collected and analyzed. The MICs of C/T and amikacin ranged from 0.008/4–128/4 μg/mL to 0.016–256 μg/mL, respectively. The results were interpreted according to the criteria of the Clinical and Laboratory Standards Institute (CLSI) [32]. All CRPA strains were stored at −80 °C until analysis. *P. aeruginosa* ATCC 27853 was used as a reference strain for quality control.

MIC values, MIC_50_, and MIC_90_ were measured for a tested population. MIC_50_ and MIC_90_ are defined as the MIC values inhibiting 50% and 90% of the tested isolates, respectively.

### 4.2. Synergy Test of C/T against CRPA

We designed a synergy test for C/T with amikacin against CRPA. The MIC values of each studied antibiotic were initially determined in order to further perform synergistic testing. For the synergy test, two E-test strips of studied antibiotics crossing formation in each MIC value with 90° angle were placed on an inoculated Mueller–Hinton Agar plate with bacteria spread. The resulting ellipse of inhibition was checked after 16–18 h at 35 ± 2 °C.

The fractional inhibitory concentration index (FICI) was calculated for each antibiotic in each combination using the following formula: FICA + FICB = ∑FICI, where FICA equals the MIC of drug A in combination divided by the MIC of drug A alone, and FICB equals the MIC of drug B in combination divided by the MIC of drug B alone. The ∑FICI were interpreted as follows: synergy, FICI ≤ 0.5; additive effect, FICI > 0.5–≤ 1; no interaction (indifference), FICI > 1–≤ 4; or antagonism, FICI > 4 [33,34].

### 4.3. Phenotypic Classification

The CRPA isolates were classified as MDR (resistant to at least one agent in three or more antimicrobial categories), XDR (resistant to at least one agent in all but two or fewer antimicrobial categories), or PDR (resistant to all agents in all antimicrobial categories) based on the CLSI criteria described by Magiorakos et al. [35].

### 4.4. Antibiotic Dosing Regimen Simulations

We used two-compartment pharmacokinetic models of C/T and amikacin with linear elimination to simulate the plasma concentration time [28,36]. Pharmacokinetic parameters, the PK/PD targets, and indices for the simulation are described in Table 8.

Simulated dosing regimens of C/T with log-normal distributions included intermittent infusion (1.5 g infusion over 0.5 h every 8 h) and extended infusion (1.5 g infusion over 4 h every 8 h and 1.5 g loading dose over 0.5 h, followed by 4.5 g continuous infusion over 24 h). The simulated dosing regimens of amikacin with log-normal distributions included a loading dose of 20–25 mg/kg, followed by maintenance doses of 15–20 mg/kg every 24 h.

The optimal antibiotic dosing regimens were simulated using a 10,000-subject Monte Carlo simulation (Oracle Crystal Ball Classroom Faculty Edition-Oracle 1-Click Crystal Ball 201, Thailand). PK/PD targets were set for each studied antimicrobial. The percentages of targets for the duration of time (*f*T) that the free drug concentration had to remain above the MIC (*f*T > MIC) were 75% and 100% for ceftolozane, and the target for tazobactam was 20% *f*T ≥ 1 µg/mL. The target ratio between the maximum drug concentration obtained after a single dose and the MIC (C_max_/MIC) was ≥ 8 for amikacin.

The probability of target attainment (PTA) was determined as the percentage of probability at which the pharmacodynamic indices with specific MICs were achieved. The cumulative fraction of response (CFR) was calculated as the proportion of % PTA for each MIC of each of the pharmacodynamic indices according to the MIC distribution [37]. At least 90% PTA at a steady state for documented therapy and 90% CFR were considered the achievement targets for empirical therapy.

## 5. Conclusions

The CRPA clinical isolates from Thailand in our study showed high in vitro susceptibility (86.36%) to C/T. Furthermore, a 40% synergistic effect was observed in combination with amikacin. C/T extended infusion regimens may be considered empirical or definite antibiotic therapies when CRPA is suspected or detected. Amikacin with a loading dose of 20 mg/kg, followed by 15 mg/kg/day, seems an attractive combination agent with C/T when combination therapy is necessary.

## Figures and Tables

**Table 1:** In vitro susceptibility and percentage of susceptibility among ceftolozane/tazobactam (C/T) and comparator agents against clinical isolates (*n* = 66) of carbapenem-resistant *Pseudomonas aeruginosa* (CRPA) from the broth micro dilution method.

Agents	MIC Range (µg/mL)	MIC_50_ (µg/mL)	MIC_90_ (µg/mL)	Percentage of Susceptible Strains ^a^
Ceftazidime	1–>32	8	>32	63.64
Cefepime	1–>32	8	>32	63.64
Piperacillin/tazobactam	8/4–>64/4	32/4	>64/4	45.45
Imipenem	≤0.5–>8	>8	>8	4.55
Meropenem	0.5–>8	8	>8	13.64
Ciprofloxacin	0.06–>2	0.25	>2	66.67
Levofloxacin	0.06–>8	2	>8	51.52
Gentamicin	2–>8	2	>8	83.33
Amikacin	8–>32	8	32	89.39
Colistin	1–>4	1	2	96.97

Abbreviations: MIC, minimum inhibitory concentration; MIC_50_, minimum inhibitory concentration required to inhibit the growth of 50% of organisms; MIC_90_, minimum inhibitory concentration required to inhibit the growth of 90% of organisms. ^a^ *Pseudomonas aeruginosa* strains are defined susceptible to the studied antibiotics and intermediate to colistin following the Clinical & Laboratory Standards Institute (CLSI) 2021. The cut-off for the susceptible breakpoint were ≤8 µg/mL for ceftazidime and cefepime, ≤16/4 µg/mL for piperacillin/tazobactam, ≤2 µg/mL for imipenem and meropenem, ≤0.5 µg/mL for ciprofloxacin, ≤1 µg/mL for levofloxacin, ≤4 µg/mL for gentamicin, ≤16 µg/mL for amikacin. The cut-off for the intermediate MIC breakpoint was ≤2 µg/mL for colistin.

**Table 2:** E-test results of antibiotic susceptibility to ceftolozane/tazobactam (C/T) (*n* = 66) and synergy testing of C/T with amikacin (*n* = 60) for all carbapenem-resistant *Pseudomonas aeruginosa* (CRPA) isolates.

No.	MIC ^a^ (µg/mL)				Synergistic Testing Results	
	C/T ^b^	AMK	C/T ^b^ Combined with AMK	AMK Combined with C/T ^b^	ΣFICI	Interpretation
1	0.50	4.00	0.38	0.75	0.94	ADD
2	1.50	3.00	0.75	0.5	0.66	ADD
3	1.00	0.50	0.38	0.094	0.56	ADD
4	0.38	3.00	0.38	1	1.33	IND
5	0.75	1.50	0.25	0.19	0.46	SYN
6	0.38	1.50	0.5	0.38	1.56	IND
7	0.25	2.00	0.25	0.75	1.37	IND
8	0.50	2.00	0.38	0.5	1.01	IND
9	1.00	3.00	0.25	0.19	0.31	SYN
10	1.50	2.00	0.75	0.38	0.69	ADD
11	0.50	4.00	0.5	4	2.00	IND
12	2.00	2.00	1	0.38	0.69	ADD
13	1.00	3.00	1	0.75	1.25	IND
14	0.75	8.00	0.75	4	1.50	IND
15	0.50	2.00	0.5	0.75	1.37	IND
16	0.50	2.00	0.5	0.75	1.37	IND
17	0.50	0.75	0.25	0.125	0.66	ADD
18	1.00	4.00	0.75	1	1.00	ADD
19	1.00	1.00	0.25	0.094	0.34	SYN
20	0.50	2.00	0.5	0.5	1.25	IND
21	0.75	1.00	0.25	0.19	0.52	ADD
22	2.00	4.00	0.75	0.5	0.50	SYN
23	1.00	2.00	1	0.75	1.37	IND
24	1.50	1.00	1	0.25	0.91	ADD
25	0.50	8.00	0.5	2	1.25	IND
26	1.50	8.00	0.5	1	0.45	SYN
27	0.75	1.50	0.5	0.25	0.83	ADD
28	1.50	6.00	0.5	1	0.50	SYN
29	2.00	4.00	0.38	0.25	0.25	SYN
30	2.00	2.00	0.75	0.25	0.50	SYN
31	64.00	1.50	32	0.25	0.66	ADD
32	3.00	1.50	1	0.19	0.46	SYN
33	0.19	2.00	0.19	0.5	1.25	IND
34	0.50	1.50	0.5	0.38	1.25	IND
35	0.38	2.00	0.25	0.38	0.84	ADD
36	0.19	0.75	0.19	0.75	2.00	IND
37	0.50	6.00	0.5	1.5	1.25	IND
38	0.75	4.00	0.25	0.5	0.45	SYN
39	0.75	2.00	0.19	0.25	0.37	SYN
40	0.25	1.00	0.125	0.19	0.69	ADD
41	0.75	1.50	0.19	0.125	0.33	SYN
42	0.75	6.00	0.25	1	0.50	SYN
43	0.38	1.50	0.19	0.25	0.66	ADD
44	0.75	3.00	0.19	0.38	0.38	SYN
45	0.50	1.00	0.5	0.25	1.25	IND
46	1.50	6.00	0.5	0.75	0.45	SYN
47	24.00	32.00	24	8	1.25	IND
48	0.75	3.00	0.094	0.19	0.18	SYN
49	0.50	3.00	0.094	0.19	0.25	SYN
50	4.00	12.00	1.5	1	0.45	SYN
51	4.00	6.00	2	1	0.66	ADD
52	0.75	1.50	0.5	0.38	0.92	ADD
53	0.75	1.50	0.25	0.19	0.46	SYN
54	1.00	3.00	0.25	0.19	0.31	SYN
55	1.50	6.00	0.38	0.5	0.33	SYN
56	8.00	6.00	2	0.5	0.33	SYN
57	4.00	3.00	1.5	0.5	0.54	ADD
58	1.50	6.00	0.5	0.75	0.45	SYN
59	0.75	3.00	0.5	0.75	0.91	ADD
60	1.00	6.00	0.38	0.75	0.50	SYN
61	>256	96.00	N/A	N/A	N/A	N/A
62	>256	12.00	N/A	N/A	N/A	N/A
63	>256	3.00	N/A	N/A	N/A	N/A
64	>256	>256	N/A	N/A	N/A	N/A
65	>256	3.00	N/A	N/A	N/A	N/A
66	>256	12.00	N/A	N/A	N/A	N/A
MIC range (µg/mL)	0.19–>256	0.5–>256	0.094–32	0.094–8	-	-
MIC_50_ (µg/mL)	0.75	3	0.5	0.5	-	-
MIC_90_ (µg/mL)	24	8	1	1	-	-
%S ^c^	86.36	95.45	96.67	100	-	-

Abbreviations: MIC, minimum inhibitory concentration; MIC_50_, MIC required to inhibit the growth of 50% of organisms; MIC_90_, MIC required to inhibit the growth of 90% of organisms; %S, percentage of susceptible strains; AMK, amikacin; C/T, ceftolozane/tazobactam; FICI, fractional inhibitory concentration index; SYN, synergistic effect (FICI ≤ 0.5); ADD; additive effect (FICI > 0.5–≤ 1); IND, indifference (FICI > 1–≤ 4); N/A, not-available. ^a^ The susceptibility testing was performed using the E-test method. ^b^ The MIC values of ceftolozane were combined with fixed tazobactam concentration (4 µg/mL). ^c^ The cut-off for the susceptible breakpoint for *Pseudomonas aeruginosa* strains following the Clinical & Laboratory Standards Institute (CLSI) 2021 were ≤4/4 µg/mL for ceftolozane/tazobactam and ≤16 µg/mL for amikacin.

**Table 3:** In vitro synergistic testing of ceftolozane/tazobactam (C/T) with amikacin against CRPA isolates (*n* = 60).

Antibiotic Combination	No (%)			
	Synergism	Additive Effect	Indifference	Antagonism
C/T + AMK	24 (40%)	18 (30%)	18 (30%)	0 (0%)

Abbreviations: AMK, amikacin; C/T, ceftolozane/tazobactam.

**Table 4:** The probability of target attainment (PTA), including 75% *f*T > MIC for ceftolozane and 20% *f*T ≥ 1 µg/mL for tazobactam, at specific MICs for each dosing regimen.

Dosage Regimens of C/T			PTA (%)												
			Ceftolozane MICs (µg/mL)												Tazo-Bactam
LD	MD	IT	0.0625	0.125	0.25	0.5	1	2	4	8	16	32	64	128	20% *f*T ≥ 1 µg/mL
-	1.5 g q 8 h	0.5 h	100.00	99.99	99.85	99.59	98.34	93.92	80.64	50.97	14.09	1.14	0.01	0.00	97.50
-	1.5 g q 8 h	4 h	100.00	100.00	100.00	100.00	99.87	98.97	93.61	69.46	23.10	1.53	0.00	0.00	97.71
1.5 g	4.5 g	CI	100.00	100.00	100.00	100.00	100.00	100.00	99.95	93.61	44.75	3.83	0.05	0.00	99.97

Abbreviations: C/T, ceftolozane/tazobactam; PTA, the probability of target attainment; MIC, minimum inhibitory concentration; LD, loading dose; MD, maintenance dose; IT, infusion time; g, gram; h, hours; q, every; CI, continuous infusion over 24 h.

**Table 5:** The probability of target attainment (PTA), including 100% *f*T > MIC for ceftolozane and 20% *f*T ≥ 1 µg/mL for tazobactam, at specific MICs for each dosing regimen.

Dosage Regimens of C/T			PTA (%)												
			Ceftolozane MICs (µg/mL)												Tazo-Bactam
LD	MD	IT	0.0625	0.125	0.25	0.5	1	2	4	8	16	32	64	128	20% *f*T ≥ 1 µg/mL
-	1.5 g q 8 h	0.5 h	99.93	99.81	99.46	98.39	95.50	87.36	69.12	38.63	8.89	0.57	0.00	0.00	97.50
-	1.5 g q 8 h	4 h	100.00	100.00	99.95	99.68	98.58	94.75	82.35	52.07	14.56	0.81	0.00	0.00	97.71
1.5 g	4.5 g	CI	100.00	100.00	100.00	100.00	100.00	100.00	99.95	93.60	44.71	3.82	0.05	0.00	99.97

Abbreviations: C/T, ceftolozane/tazobactam; PTA, the probability of target attainment; MIC, minimum inhibitory concentration; LD, loading dose; MD, maintenance dose; IT, infusion time; g, gram; h, hours; q, every; CI, continuous infusion over 24 h.

**Table 6:** The cumulative fraction of response (CFR) for each dosing regimen of ceftolozane/tazobactam (C/T) and ceftolozane/tazobactam (C/T), combined with amikacin (AMK).

Dosage Regimens of C/T			CFR (%)			
LD	MD	IT	75% *f*T > MIC		100% *f*T > MIC	
			C/T	C/T Combined with AMK	C/T	C/T Combined with AMK
-	1.5 g q 8 h	0.5 h	84.24	95.79	80.93	94.24
-	1.5 g q 8 h	4 h	86.82	96.62	84.61	95.94
1.5 g	4.5 g	CI	87.84	96.79	87.84	96.79

Abbreviations: C/T, ceftolozane/tazobactam; AMK, amikacin; CFR, cumulative fraction of response; MIC, minimum inhibitory concentration; LD, loading dose; MD, maintenance dose; IT, infusion time; g, gram; h, hours; q, every; CI, continuous infusion over 24 h.

**Table 7:** The cumulative fraction of response (CFR) for each dosing regimen of amikacin (AMK) and amikacin (AMK) combined with ceftolozane/tazobactam (C/T).

Dosage Regimens of AMK			CFR (%)	
LD	MD	IT	C_max_/MIC ≥ 8	
			AMK	AMK Combined with C/T
20 mg/kg	15 mg/kg q 24 h	0.5 h	62.52	97.30
25 mg/kg	15 mg/kg q 24 h	0.5 h	62.79	97.36
25 mg/kg	20 mg/kg q 24 h	0.5 h	67.08	98.03

Abbreviations: C/T, ceftolozane/tazobactam; AMK, amikacin; CFR, cumulative fraction of response; MIC, minimum inhibitory concentration; LD, loading dose; MD, maintenance dose; IT, infusion time; mg, milligram; kg, kilogram; h, hours; q, every.

**Table 8:** Pharmacokinetic parameters, the PK/PD targets, and indices of ceftolozane/tazobactam (C/T) and amikacin used for the simulation.

Antibiotics	Parameters	Mean	SD	%RSE	PK/PD Targets and Indices
Ceftolozane	V (L)	20.4	3.7	-	75% *f*T > MIC, 100% *f*T > MIC
	Kcp (h^−1^)	0.46	0.74	-	
	Kpc (h^−1^)	0.39	0.37	-	
	CL (L/h)	7.2	3.2	-	
Tazobactam	V (L)	32.4	10	-	20% *f*T ≥ 1 µg/mL
	Kcp (h^−1^)	2.96	8.69	-	
	Kpc (h^−1^)	26.5	8.4	-	
	CL (L/h)	25.4	9.4	-	
Amikacin	CL (L/h)	0.77	-	28.4	C_max_/MIC ≥ 8
	V (L)	19.2	-	5.31	
	Q (L/h)	4.38	-	18.3	
	V_p_ (L)	9.38	-	7.15	

Abbreviations: V, typical volume of distribution of the central compartment; Vp, peripheral volume of distribution; Kcp, rate constant for distribution of unbound ceftolozane or tazobactam from central to peripheral compartment; Kpc, rate constant for distribution of unbound ceftolozane or tazobactam from peripheral to central compartment; CL, clearance; SD, standard deviation; PK/PD, pharmacokinetics/pharmacodynamics; MIC, minimum inhibitory concentration; RSE, relative standard error.

## Data Availability

Data available on request due to restrictions.
